# Evaluation of Anti-A/Udorn/307/1972 Antibody Specificity to Influenza A/H3N2 Viruses Using an Evanescent-Field Coupled Waveguide-Mode Sensor

**DOI:** 10.1371/journal.pone.0081396

**Published:** 2013-12-10

**Authors:** Subash C. B. Gopinath, Koichi Awazu, Makoto Fujimaki, Kazufumi Shimizu

**Affiliations:** 1 Electronics and Photonics Research Institute, National Institute of Advanced Industrial Science and Technology, Central 5, Tsukuba, Ibaraki, Japan; 2 Open Research Center for Genome and Infectious Disease Control, Nihon University School of Medicine, Itabashi-ku, Tokyo, Japan; Nihon University School of Medicine, Japan

## Abstract

Discrimination of closely related strains is a key issue, particularly for infectious diseases whose incidence fluctuates according to variations in the season and evolutionary changes. Among infectious diseases, influenza viral infections are a worldwide cause of pandemic disease and mortality. With the emergence of different influenza strains, it is vital to develop a method using antibodies that can differentiate between viral types and subtypes. Ideally, such a system would also be user friendly. In this study, a polyclonal antibody generated against A/Udorn/307/1972 (H3N2) was used as a probe to distinguish between influenza H3N2 viruses based on the interaction between the antibody and hemagglutinin, demonstrating its applicability for viral discrimination. Clear discrimination was demonstrated using an evanescent-field-coupled waveguide-mode sensor, which has appealing characteristics over other methods in the viewpoint of improving the sensitivity, measurement time, portability and usability. Further supporting evidence was obtained using enzyme-linked immunosorbent assays, hemagglutination-inhibition assays, and infectivity neutralization assays. The results obtained indicate that the polyclonal antibody used here is a potential probe for distinguishing influenza viruses and, with the aid of a handheld sensor it could be used for influenza surveillance.

## Introduction

Influenza viruses are spherical or filamentous, enveloped, and range in size from 80 to 100 nm [1]. Influenza is a pandemic disease caused by different viral strains, which emerge during seasonal changes. Influenza viruses belong to the Orthromyxoviridae family and can be classified into 3 types, namely, A, B and C. Influenza A viruses are further divided into subtypes based on their envelope proteins, that is, hemagglutinin (HA) and neuraminidase (NA); to date, 16 types of HA and 9 types of NA have been identified, and recently, a new HA (H17) and NA (N10), divergent from all known influenza HAs and NAs, respectively, were found in the little yellow-shouldered bat [2], [3]. Influenza B viruses are classified into the Yamagata and Victoria lineages [4]. These classifications, based on types and subtypes, are vital for differentiating the emerging new strains from older ones; for example, the recently emerged influenza pandemic virus was named A(H1N1)pdm09.

To detect these different strains, a probe for detecting small variations in HA or NA is required. Among the probes developed for recognizing different HA or NA molecules, antibodies are very commonly used, and have been generated against different strains for the eventual purpose of influenza discrimination. To pinpoint differences among influenza H3N2 viruses by using a polyclonal antibody as the probe, we developed a detection method using an anti-A/Udorn/307/1972 polyclonal antibody on an evanescent-field-coupled waveguide-mode (EFC-WM) sensor. Evidence of the effectiveness of this antibody in distinguishing between strains has been supported by the results of commonly used biological assays.

Several antibody-based immunoassays and immunosensors have been generated with a view to future applications [5]–[12]. Antibody-based sensors permit rapid and sensitive analyses for a wide range of biomolecules, including pathogens and associated toxins [13]. These assays or sensors are ligand-analyte–based designs that involve antigen-antibody binding. This model is also used in the EFC-WM sensor described here, which is based on a principle similar to that of the common surface plasmon resonance (SPR) sensing system; the difference is that the EFC-WM sensor uses waveguide modes, instead of SPR [14]–[22]. In the case of the SPR sensor, the wavelength of incident light is restricted by the material used to induce the SPR, whereas there is no such restriction for the EFC-WM sensor. In addition, a stable sensing surface made of glass is available in the case of the EFC-WM sensor. Another advantage of the EFC-WM sensor over current sensors is that higher sensitivity can be easily obtained using biomolecules labeled with dyes or metal nanoparticles. The EFC-WM sensor has sensitivity equal to enzyme-linked immunosorbent assay (ELISA), whereas the EFC-WM sensor consumes lesser experimental time than ELISA [6], [20]–[21], [23].

In the present study, in order to enhance sensitivity, an anti-A/Udorn/307/1972 antibody conjugated with gold nanoparticles (AuNPs) was employed as performed before [23]. We have previously demonstrated the antibody-based detection of human and avian influenza viruses using the EFC-WM sensor and the detection limit of this sensor was determined to be in the order of 10^3^ pfu/ml [6], [23]. The present study explores a further step, using antibody-based sensing for discrimination of influenza viruses belonging to the H3N2 subtype.

## Results and Discussion

**Figure 1:**
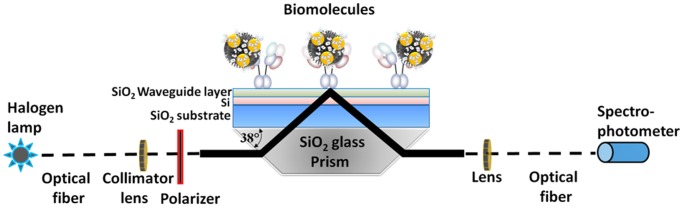
Diagrammatic representation of a compact EFC-WM sensor system (light source, halogen lamp).

Understanding closely related species or molecules is important for the purposes of functional analyses and diagnosis. In the case of diagnosis, various sensing systems have been proposed to discriminate biological molecules using a single probe and have had a great impact. In the past, a high degree of discrimination using a single probe, with several thousand-fold differences between molecules, has also been proposed [24]–[27]. For clinical applications, distinguishing between biological strains that cause diseases is an important issue for preventing the spread of the disease and consequent mortality. Influenza is a pandemic disease, which is widespread worldwide and is a critical threat for human health and the economy. There is an urgent requirement for developing a sensing system that can accurately discriminate between different influenza viruses. Generally influenza strains are classified based on the surface antigens HA and NA; these molecules mediate interactions with host cells during different stages of infection. Different anti-HA probes are available for detection of viral infections; these have been implemented in several diagnostic methods, including enzyme-linked immunosorbent assays, immunoblots, immunosensor-based methods, interferometry, fluoroimmunoassays, methods based on SPR, and immunochromatography [6]–[11], [26], [28]. Because HA is a major determinant in the causation of epidemics [29], the majority of these diagnostic systems use an anti-HA antibody as the probe. Herein, we demonstrate a method for detecting and discriminating influenza A/H3N2 viruses, using the EFC-WM sensor (Figure 1). To produce an antibody for accurate discrimination, we immunized healthy rabbits using A/Udorn/307/1972 and obtained an anti-A/Udorn/307/1972 polyclonal antibody.

### ELISA

**Figure 2:**
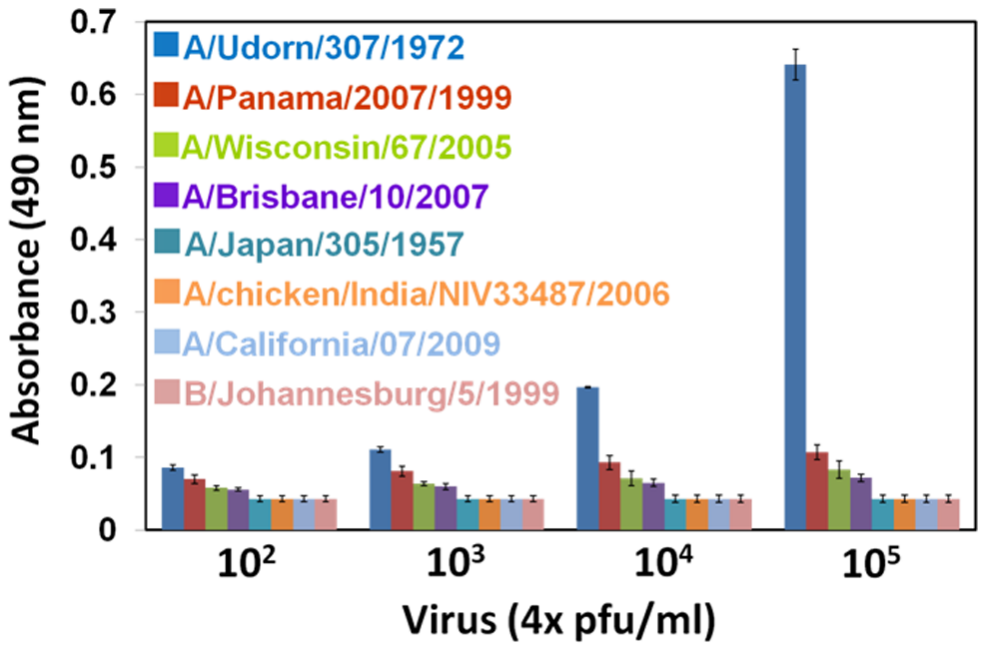
ELISA analyses of the viruses against the polyclonal antibody. Different concentrations of the viruses are titrated; 150 ng/ml of the polyclonal antibody was used. For detection, 10 µg/ml of HRP-conjugated anti-rabbit IgG was used as a secondary antibody. *o*-Phenylenediamine dihydrochloride substrate (0.5 mg/ml) dissolved in hydrogen peroxide substrate buffer was used for the color development and measured at 490 nm.

Initially, we confirmed the predominant binding of A/Udorn/307/1972 and the polyclonal antibody by ELISA, as this is currently considered as the “gold standard” assay for several infectious diseases [30], [31]. The ELISA demonstrated dose-dependent binding of A/Udorn/307/1972 to the polyclonal antibody. As a comparison, 3 other H3N2 strains, A/Panama/2007/1999, A/Wisconsin/67/2005, and A/Brisbane/10/2007, were also examined. The polyclonal antibody showed less affinity with these 3 strains (Figure 2). In addition, HAs from other subtype influenza viruses, A/Japan/305/1957 (H2N2), A/chicken/India/NIV33487/2006 (H5N1), A/California/07/2009 (H1N1), and B/Johannesburg/5/1999, were examined. All 4 of these proteins completely failed to bind the polyclonal antibody, showing only background levels of absorbance (Table 1).

**Table 1:** Consolidated data from the assays for evaluating the interactions of influenza viruses and the polyclonal antibody.

	Influenza A							Influenza B
	*H3N2*				*H2N2*	*H5N1*	*H1N1*	*Yamagata*
Assay	Udorn	Panama	Wisconsin	Brisbane	Japan	India	California	Johannesburg
*WM*	+++	+	+	+	−	−	−	−
*ELISA*	+++	+	+	+	−	−	−	−
*HI*	+++	−	−	−	−	−	−	−
*IN*	+++	−	−	−	−	−	−	−

+++ High affinity; +low affinity; − no affinity.

WM - evanescent field-coupled waveguide-mode sensor; ELISA - enzyme-linked immunosorbent assay; HI - Hemagglutination inhibition; IN - Infectivity neutralization.

### Hemagglutination Inhibition Assay and Infectivity Neutralization Assay

**Figure 3:**
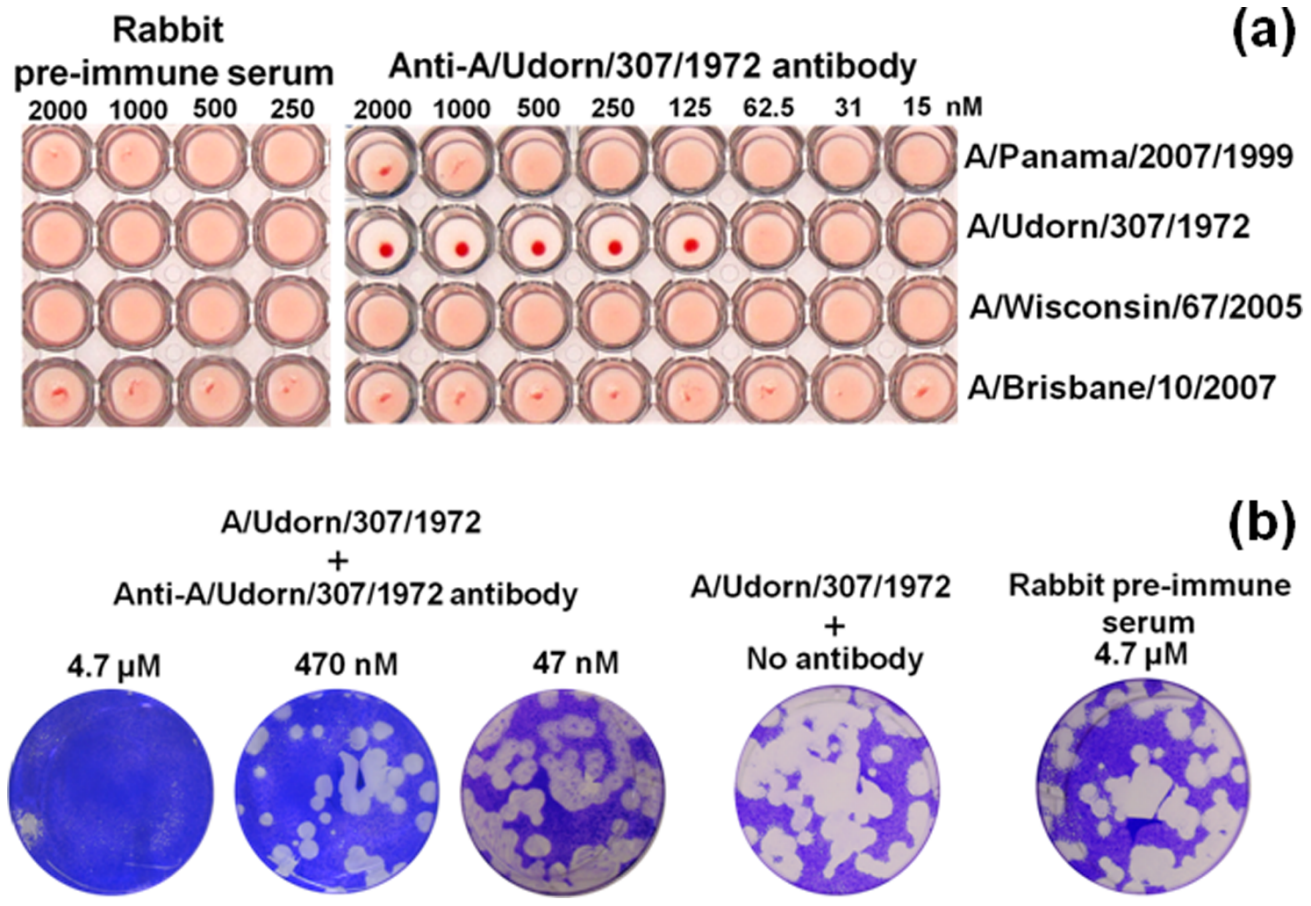
Biological assays with H3N2 viruses and the polyclonal antibody. (a) Hemagglutination assay. Agglutination assays were conducted in 96-well, plastic, round bottomed plates in the presence of 0.25% chicken red blood cells. Formation of a hazy layer in the reaction mix indicates agglutination. Agglutination was evaluated with the viruses, both in the presence or absence of anti-A/Udorn/307/1972 antibody. Rabbit pre-immune serum was used for control reactions. (b) Neutralization assay. Assays were performed in 6-well culture plates seeded with MDCK cells. To evaluate the neutralization ability of the polyclonal antibody, 47 nM, 470 nM, and 4.7 µM of the antibody was mixed with A/Udorn/307/1972 viruses (100 plaques).

The binding abilities of the polyclonal antibody to the homologous strains were further supported by 2 biological assays, hemagglutination inhibition and infectivity neutralization assays. These assays were used to assess the function of surface antigens of influenza viruses [32], [33]. The 4 H3N2 strains listed above were examined. In the hemagglutination inhibition assay, 8 HA units were chosen for all the influenza viruses. We observed agglutination reactions for all viruses, except for A/Udorn/307/1972, in the presence of the polyclonal antibody. In the case of A/Udorn/307/1972, agglutination was prevented by the polyclonal antibody with a concentration of 125 nM or higher. The blockage of hemagglutination inhibition by the polyclonal antibody at lower dilutions indicated its specificity. As a control reaction, the pre-immune serum from the same rabbit used for the antibody generation was used instead of the polyclonal antibody. In this case, agglutination was observed for all the viruses even with higher serum concentrations than those shown the inhibition with polyclonal antibody (Figure 3a; Table 1).

All 4 H3N2 viruses were also used for the neutralization assay. Madin-Darby canine kidney (MCDK) cells were cultured and infected with the viruses in the presence or absence of the polyclonal antibody. Neutralization was not observed in the case of Udorn/307/1972 in the absence of antibody (pre-immune serum control), whereas neutralization was observed by adding the polyclonal antibody (Figure 3b). The antibody did not have a neutralization effect on the other viruses tested (Table 1). These bioassay results indicate the predominant affinity of the polyclonal antibody against A/Udorn/307/1972, and therefore, effective detection and discrimination of the virus were expected when using the EFC-WM sensor with the polyclonal antibody.

### Discrimination of H3N2 Influenza Viruses by the EFC-WM Sensor

**Figure 4:**
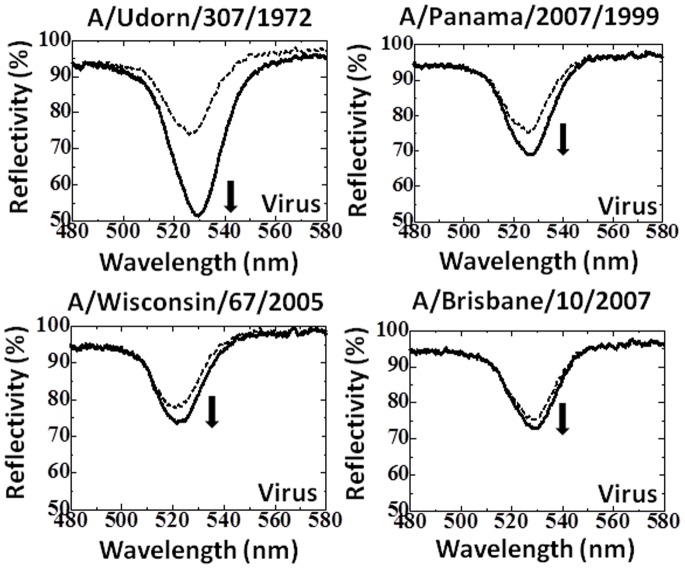
Spectra showing the interactions of the immuno-AuNPs and H3N2 viruses. Here, 8×10^5^ pfu/ml of viruses treated with 0.5% Triton X-100 were applied on the sensing plate and immobilized by the capture antibody. The dotted and bold lines represent the reflection spectra measured after the attachment of the viruses and after the attachment of immuno-AuNPs, respectively.

**Figure 5:**
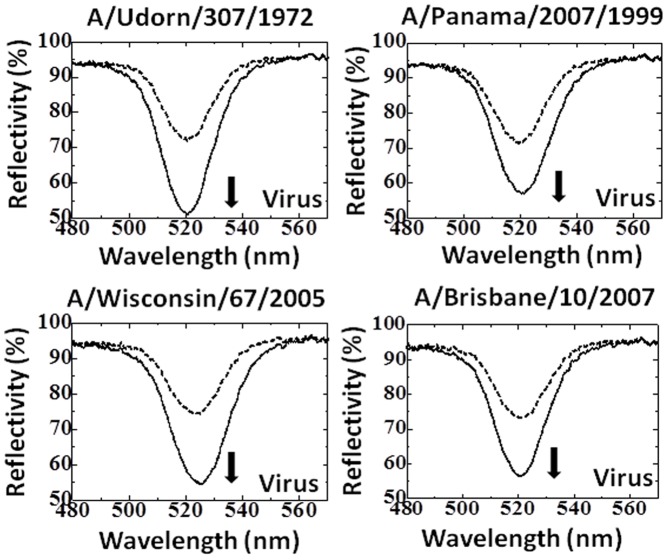
Spectra showing the interactions of the anti-H3N2 monoclonal antibody (MAb) conjugated AuNPs and H3N2 viruses. Here, 8×10^5^ pfu/ml of viruses treated with 0.5% Triton X-100 were applied on the sensing plate and immobilized by the capture MAb. The dotted and bold lines represent the reflection spectra measured after the attachment of the viruses and after the attachment of the MAb conjugated AuNPs, respectively.

We used the polyclonal antibody immobilized on EFC-WM sensing plates as the capture molecule and the captured viruses were detected by adding immuno-AuNP (AuNP conjugated with the polyclonal antibody). Initially, instead of the polyclonal antibody, the pre-immune serum was tested as the capture molecule on the EFC-WM sensor with the A/Udorn/307/1972 virus and immuno-AuNP binding. No binding was observed (data not shown). In order to examine the detection and discrimination ability of the polyclonal antibody, we tested the reactivity of the 4 H3N2 viruses (8×10^5^ pfu/ml) with the antibody using the EFC-WM sensor. The binding of the viruses was measured using the immuno-AuNP, with 1.5 optical density (O.D.), which did not result in non-specific binding. Changes in reflection spectra obtained by the EFC-WM sensor were measured, and a significant change in the spectra was observed with A/Udorn/307/1972, with a change in reflectivity of more than 20%. On the other hand, in the case of the other viruses, the reflectivity changes were small, indicating a weaker binding affinity (Figure 4). As a comparison, a monoclonal antibody (MAb) against recent H3N2 viruses was used for the capture and detection antibodies, where the detection antibody was conjugated with AuNP and evaluated against the above tested viruses. The MAb showed high binding affinity with all the H3N2 viruses tested (Figure 5). The above results demonstrate the predominant binding of the polyclonal antibody with A/Udorn/307/1972, which allows the antibody to distinguish A/Udorn/307/1972 from other influenza viruses, indicating the specific interaction of anti-A/Udorn/307/1972 antibody with A/Udorn/307/1972.

### Predominant Binding of Hemagglutinin from A/Udorn/307/1972

**Figure 6:**
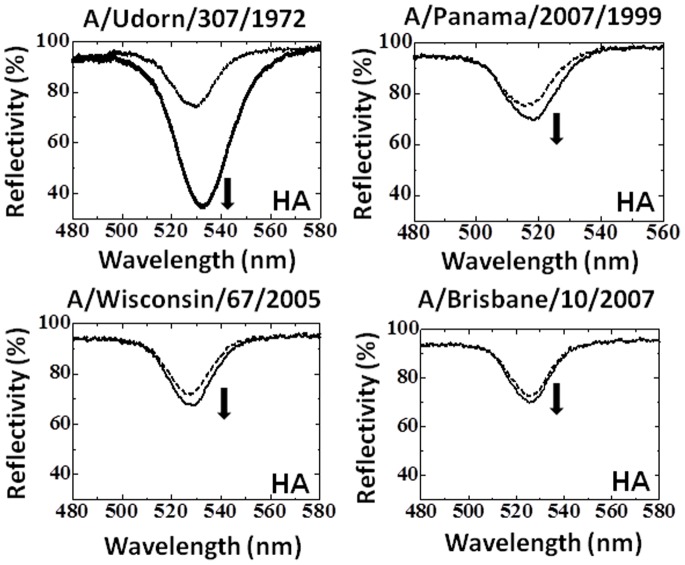
Spectra showing the interactions of the immuno-AuNPs and the HAs of 4 H3N2 viruses. The viruses used were A/Udorn/307/1972, A/Panama/2007/1999, A/Wisconsin/67/2005, and A/Brisbane/10/2007. The concentration of the HAs examined was 50 nM. The HAs were immobilized by the capture antibody. The dotted and bold lines represent the reflection spectra measured after the attachment of the HAs and after the attachment of immuno-AuNPs, respectively.

It has been reported that each viral particle is covered with ∼400 copies of the HA trimer [29], which has dominant antigenicity. In order to confirm that the polyclonal antibody recognized the HA molecule, we performed binding analyses between the polyclonal antibody and the HAs using the EFC-WM sensor. A purified HA from A/Udorn/307/1972 was used in this experiment. As described above, the polyclonal antibody was used as the capture molecule and the same antibody conjugated with AuNP was used as the detection molecule. A signal indicating good binding affinity was observed using 50 nM HA, with reflectivity changes of 40% (Figure 6). The binding of the polyclonal antibody with HAs from the other 3 influenza strains, A/Panama/2007/1999, A/Wisconsin/67/2005, and A/Brisbane/10/2007 was weaker, indicating that the antibody’s binding affinity with these proteins was much less than that with the HA of A/Udorn/307/1972. These results indicate that the polyclonal antibody is highly specific for the HA of A/Udorn/307/1972 and that the observed binding to the whole virus is predominantly through the HA.

**Figure 7:**
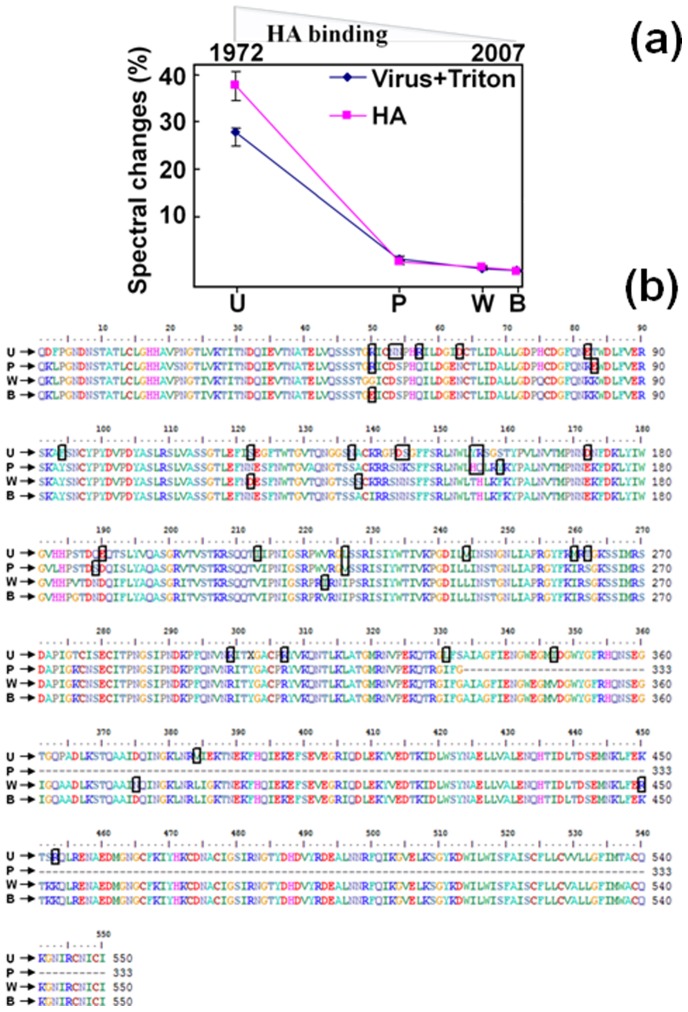
Graphical representation showing changes in reflectivity induced by the binding of the H3N2 viruses and the HAs of H3N2 viruses against the polyclonal antibody is shown (a). The x-axis represents the year in which the viruses emerged. The symbols, U, P, W, and B represent A/Udorn/307/1972, A/Panama/2007/1999, A/Wisconsin/67/2005, and A/Brisbane/10/2007, respectively. b, shows Amino acid sequence identities of the HAs from the 4 tested influenza H3N2 viruses. The signal-peptide regions are not considered and sequences are aligned with gaps. Regions of amino acid variation are indicated with boxes.

**Figure 8:**
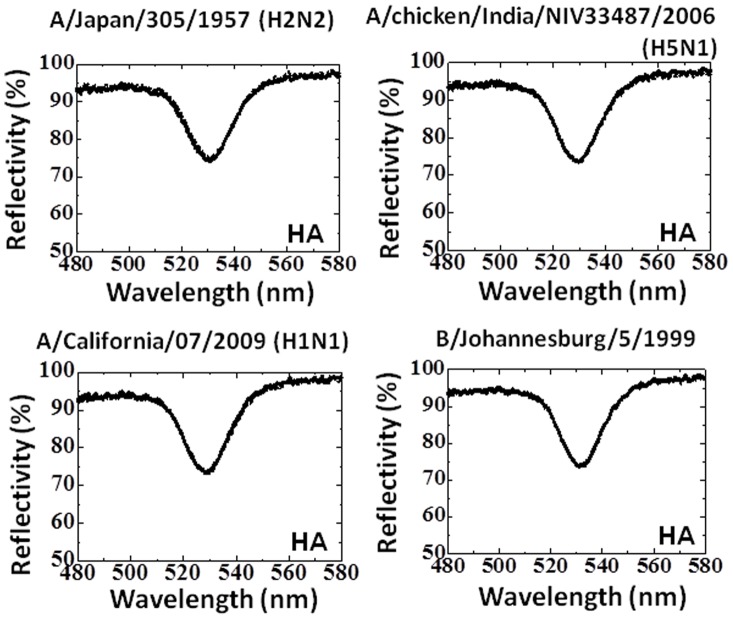
Spectra showing the interactions of the immuno-AuNPs and the HAs of viruses other than H3N2. The concentration of the HAs examined was 50-AuNPs, respectively.

Notably, viruses emerged later than A/Udorn/307/1972 and their HA molecules show lesser affinity with the polyclonal antibody generated against A/Udorn/307/1972 (Figure 7a). Based on the present experiments, we could not specify the epitope for anti-A/Udorn/307/1972 antibody. The amino acid regions from H3N2 viruses were revealed by sequence alignment among the HAs, and the possible epitopes are the regions including the parts indicated by boxes (Figure 7b). Further, we tested HAs from other subtypes of influenza viruses, including recently-characterized H1N1 (seasonal and 2009 pandemic) and type B, it confirmed that the polyclonal antibody had no affinity with A/Japan/305/1957 (H2N2), A/chicken/India/NIV33487/2006 (H5N1), A/California/07/2009 (H1N1), and B/Johannesburg/5/1999 on EFC-WM sensor (Figure 8). The polyclonal antibody shown here may be an additional candidate for differentiating influenza viruses, along with other proven probes. Development of this type of antibody would speed up the process of surveillance for emerging influenza strains.

This study demonstrated that the anti-A/Udorn/307/1972 polyclonal antibody has the ability to discriminate between old and recently emerged influenza A/H3N2 viruses. In addition, the results obtained by the EFC-WM sensor imply that the generation of influenza A/H3N2 viruses can be estimated on the basis of signal intensity. These analyses anchor the applicability of polyclonal antibody on EFC-WM sensor for the diagnosis of clinical relevant samples and to discriminate the emergence of different influenza strains. The detection time on EFC-WM sensor is shorter than the time required for ELISA and the sensitivity of EFC-WM sensor is better than immunochromatography test.

## Materials and Methods

### Reagents and Biomolecules

We purchased CDI, crystal violet, and phosphate-buffered saline from Wako Chemicals (Osaka, Japan). The H3N2 viruses, including A/Panama/2007/1999, A/Wisconsin/67/2005, and A/Brisbane/10/2007, and recombinant HA of A/Wisconsin/67/2005, were from Prospec-Tany TechnoGene Ltd. (Rehovot, Israel). Recombinant HAs of A/Panama/2007/1999, and A/Brisbane/10/2007 were from Immune Technology Corporation (New York, USA). Recombinant HAs from avian influenza virus H2N2 (A/Japan/305/1957), H5N1 (A/chicken/India/NIV33487/2006), and H1N1 (A/California/07/2009), were from Sino Biological Inc. (Beijing, China). The mouse anti-influenza A (H3N2) monoclonal antibody was from Millipore (CA, USA). Horseradish peroxidase-conjugated anti-rabbit IgG was obtained from Promega (Madison, USA). The ELISA titer plate was purchased from Costar, Corning Incorporated (NY, USA); bovine serum albumin from Sigma Chemicals (MO, USA); and the ELISA coating buffer was from Biolegend (San Diego, USA). *o*-Phenylenediamine dihydrochloride (OPD) and stable peroxide substrate buffer were from Thermo Scientific (Rockford, USA). All samples were stored according to the suppliers’ recommendations.

The anti-influenza A/Udorn/307/1972 polyclonal antibody was generated in our laboratory using the purified A/Udorn/307/1972 influenza virus by immunizing rabbits and with the aid of standard immunological procedures [34]. Serum was collected from rabbits before (pre-immune) and after (post-immune) immunization. Anti-A/Udorn/307/1972 antibody was chemically conjugated with 10-nm diameter AuNPs as described earlier [23]. Amino acid sequence alignment for HAs was performed for all tested H3N2 viruses: A/Udorn/307/1972 (GenBank accession no. AAA43099; 566 amino acids), A/Panama/2007/1999 (GenBank accession no. ABQ58929; 333 amino acids), A/Wisconsin/67/2005 (GenBank accession no. ABW80978; 566 amino acids), and A/Brisbane/10/2007 (GenBank accession no. ABW23424; 550 amino acids).

### ELISA

ELISA was performed in 96-well high affinity, flat-bottomed, polystyrene plastic plates. Initially, the viruses (50 µl) were attached using commercially obtained coating buffer (8.4 g NaHCO_3_; 3.56 g Na_2_CO_3_; ddH_2_O added up to 1.0 L, pH 9.5), and incubated at room temperature for 2 h. After washing with PBS, free surfaces were masked using 300 µl of a 2% BSA solution. Next, the polyclonal antibody was used as the primary antibody with a concentration of 150 ng/mL and incubated overnight at 4°C. Thorough washing steps were then carried out using PBS containing 0.05% Tween 20, followed by incubation with 50 µl of anti-rabbit IgG conjugated with horseradish peroxidase (HRP) with a concentration of 10 µg/ml. After incubation for 1 h at room temperature, thorough washing with PBS containing 0.05% Tween 20 was performed. Utilizing the captured HRP, color development was measured with the OPD substrate (0.5 mg/ml) dissolved in hydrogen peroxide substrate buffer (0.05 M citric acid, 0.05 M sodium phosphate; pH 5; 1 µl of 30% hydrogen peroxide added per milliliter of substrate buffer). After incubation at room temperature for 30 min, the reactions were stopped by adding an equal volume of 2.5 N H_2_SO_4_. Optical density measurements (490 nm) were then carried out. All incubations were performed with agitation in plates sealed with plastic sheets.

### Hemagglutination Inhibition Assay

Two-fold serial dilutions (from 15 to 2000 nM) of the polyclonal antibody were made in 25 µl of PBS, pH 7.4, in 96-well U-bottom plates (BD, Franklin Lakes, NJ, USA). Pre-immune serum (dilutions from 250 to 2000 nM) was used as a control. Then, 25 µl of the viruses (8 HAU/ml PBS) were added for each dilution before the plates were incubated for 1 h at 37°C. To each well, 50 µl of 0.5% (v/v) chicken blood cells (Nippon Biotest Laboratories, Tokyo, Japan) in PBS was added. Initially, we confirmed that agglutination had occurred by using whole chicken blood with the above dilutions and observed the formation of clear RBC red buttons, indicating that the RBCs were intact. The reciprocal of the lowest concentration that completely inhibited hemagglutination was taken as the hemagglutination inhibition (HI) titer; 1 HAU was defined as the quantity of virus present in 1 ml of virus suspension with an HA titer of 1 [35].

### Infectivity Neutralization Assay

Viruses were propagated and the infectivity of the viruses was determined by a plaque assay as described previously [34], [35]. Viral samples were prepared in Hanks balanced salt solution (HBSS; Life Technologies, Grand Island, NY, USA) and inoculated on MDCK cell monolayers in 6-well tissue culture plates (Corning, Lowell, MA, USA). Adsorption of the virus onto the cells was carried out for 30 min at room temperature; then, 1.6 ml of Leibovitz’s L15 medium (Life Technologies) containing 0.6% SeaKem ME agarose (Lonza, Basel, Switzerland), 1.5% gelatin (Nacalai, Kyoto, Japan), and 2.5 µg/ml TPCK-trypsin was added to each well and solidified. The polyclonal antibody samples with concentrations of 47 nM, 470 nM, and 4.7 µM were mixed with an equal volume of virus suspension (approximately 1×10^4^ pfu/ml) and incubated for 1 h at 37°C. The mixtures were then titrated for infectivity by a plaque assay. Control reactions were carried out using A/Udorn/307/1972 without the polyclonal antibody and mixed with pre-immune rabbit serum (4.7 µM; calculated based on immunoglobulin molecular weight). Live and dead cells were visualized by flooding the wells with 0.5% filtered crystal violet in PBS for 20 min at room temperature, MDCK cells were washed with water for clear visualization, and the plates were photographed. The assay was performed in triplicate.

### EFC-WM Sensor System and Measurements

The EFC-WM sensor uses a sensing plate with a multilayer structure that consists of a dielectric waveguide, a high refractive index layer, and a glass substrate (Fujimaki et al., 2008). The sensing plate, illuminated under the Kretschmann configuration, operates as a sensor capable of detecting modifications in the dielectric environment near the waveguide surface by measuring changes in reflectivity. In this study, we utilized a spectral-readout-type EFC-WM sensor. In the experiment, a halogen lamp was used as a light source. The light from the lamp was guided to a collimator lens, and the collimated light was passed through a polarizer and irradiated into a prism, where the incident angle was parallel to the bottom face of the prism. Then, a sensing plate, placed on the bottom of the prism, was illuminated, and the spectrum of the reflected light was recorded using a spectrophotometer (Ocean Optics, Florida, USA). The prism was made of SiO_2_ glass, and the bottom angle of the prism was 38° (Figure 1). The sensing plate consisted of a SiO_2_ glass substrate, a single crystalline Si layer, and a thermally grown SiO_2_ waveguide. In this experiment, the thicknesses of the SiO_2_ glass waveguide and the single crystalline Si layer were 45 and 360 mm, respectively. The sensing system was adjusted to show a dip in reflectance at approximately 520 nm, which corresponds to the optical absorption of immuno-AuNPs. If AuNPs are attached to the EFC-WM sensor surface, the dip is deepened by the optical absorption of the AuNP [19].

The sensing plates were treated with 0.1 M potassium phosphate buffer, pH 9.4 for 30 min, washed thoroughly with water, and dried. Then, modification with 0.5 M CDI in dioxane of the sensing plate was performed at 37°C for 2 h, followed by rinsing with acetone and then water before being dried. On this surface, the polyclonal antibody (500 nM) was immobilized and blocked with 1 M ethanolamine (GE Healthcare, USA). The coupling reaction for CDI and the antibody was conducted for 3 h. Triton X-100 (0.5%) treated Virus or untreated HA samples were placed on this antibody-immobilized sensor surface, and change in reflection spectra were observed by attaching immuno-AuNPs (anti-A/Udorn/307/1972 polyclonal antibody conjugated AuNPs). Each incubation was performed for 30 min at room temperature. Mouse anti-influenza-A MAb against recent H3N2 strains was also coupled with 10 nm AuNPs using a previously described procedure [36] and was used for comparative analyses.

### Purification of Hemagglutinin

Purified hemagglutinin from the A/Udorn/307/1972 virus was obtained by using a previously described strategy [26]. Briefly, 250 µl of virus from the stock (8×10^9^ pfu/ml) was suspended in buffer containing 50 mM Tris, 25 mM NaCl, and 1 mM CaCl_2_, pH 7.5. This viral solution was centrifuged at 15,000 rpm for 30 min at 4°C, and the pellet was collected in order to remove the sucrose existing in the original stock. This pellet was resuspended in the buffer described above, with the addition of 4% Triton X-100 to the final reaction volume and maintained at 37°C for 2 h. After further centrifugation at 15,000 rpm for 30 min at 4°C, the supernatant was applied to a Vivapure S-type column (Sartorius AG, Germany). Next, the column was thoroughly washed with copious volumes of buffer containing 50 mM Tris, 25 mM NaCl, and 1 mM CaCl_2_, pH 7.5. The bound HA was eluted using the above buffer with 1 M NaCl.
